# Pregnancy in the time of COVID-19: towards Fetal monitoring 4.0

**DOI:** 10.1186/s12884-023-05349-3

**Published:** 2023-01-16

**Authors:** Radana Kahankova, Katerina Barnova, Rene Jaros, Jan Pavlicek, Vaclav Snasel, Radek Martinek

**Affiliations:** 1 Department of Cybernetics and Biomedical Engineering, Faculty of Electrical Engineering and Computer Science, VSB–Technical University of Ostrava, Ostrava, Czechia; 2 Department of Pediatrics, Faculty Hospital, Faculty of Medicine, Ostrava University, Ostrava, Czechia; 3 Department of Computer Science, Faculty of Electrical Engineering and Computer Science, VSB–Technical University of Ostrava, Ostrava, Czechia

**Keywords:** COVID-19, Fetal monitoring 4.0, Fetal telemonitoring, Healthcare 4.0, Non-invasive fetal electrocardiography (NI-fECG), Remote fetal monitoring, Smart healthcare

## Abstract

On the outbreak of the global COVID-19 pandemic, high-risk and vulnerable groups in the population were at particular risk of severe disease progression. Pregnant women were one of these groups. The infectious disease endangered not only the physical health of pregnant women, but also their mental well-being. Improving the mental health of pregnant women and reducing their risk of an infectious disease could be achieved by using remote home monitoring solutions. These would allow the health of the mother and fetus to be monitored from the comfort of their home, a reduction in the number of physical visits to the doctor and thereby eliminate the need for the mother to venture into high-risk public places. The most commonly used technique in clinical practice, cardiotocography, suffers from low specificity and requires skilled personnel for the examination. For that and due to the intermittent and active nature of its measurements, it is inappropriate for continuous home monitoring. The pandemic has demonstrated that the future lies in accurate remote monitoring and it is therefore vital to search for an option for fetal monitoring based on state-of-the-art technology that would provide a safe, accurate, and reliable information regarding fetal and maternal health state. In this paper, we thus provide a technical and critical review of the latest literature and on this topic to provide the readers the insights to the applications and future directions in fetal monitoring. We extensively discuss the remaining challenges and obstacles in future research and in developing the fetal monitoring in the new era of Fetal monitoring 4.0, based on the pillars of Healthcare 4.0.

## Introduction

The COVID-19 pandemic, caused by severe acute respiratory syndrome coronavirus 2 (SARS-CoV-2), has affected almost all areas of human life, including the provision of healthcare. The main challenge faced by healthcare providers has been to ensure patient safety, while maintaining the quality of the healthcare services provided [1]. Particular attention was focused on patients who had a higher risk of severe COVID-19 progression. According to the World Health Organization (WHO) [2], this risk group includes pregnant women, among others, especially those who are older, overweight, and have pre-existing medical conditions (hypertension and diabetes). However, due to the physiological changes in their body, pregnant women are a vulnerable group in the event of an outbreak of any infectious disease (even without pre-existing medical conditions).

In addition to health risks, the COVID-19 pandemic has also had a huge impact on the mental state of pregnant women, leading to stress, anxiety and depression [3–9]. Stress during pregnancy is most often associated with higher rates of preterm deliveries and the delivery of low-birth-weight babies [4, 10, 11]. Prenatal mental stress can lead to an increased risk of gestational hypertension and preeclampsia [10, 12, 13]. The impact of prenatal stress on the neuropsychological development of the fetus [10, 14] and the behavioral and physiological development of offspring [15] has also been shown.

Protecting pregnant women and their fetuses should therefore be a priority [16]. Quarantine, hygiene measures and social distancing have proved to be effective protection against the spread of COVID-19. However, if these measures are not adequately followed, there is a higher risk of contracting this viral disease [17, 18].

## Covid-19 and pregnancy

Approximately 80.0% of COVID-19 cases in pregnant women were asymptomatic or exhibited mild symptoms [19]. In mild cases of the disease, pregnant women exhibited similar symptoms to non-pregnant women. The most common symptoms were fatigue (54.5%), followed by a cough (50.1%) and fever (27.6%). Other symptoms included dyspnea (21.0%), myalgia (16.0%) and a sore throat (11.0%) [20]. In severe cases of COVID-19, pregnant women required more frequent hospitalization in an intensive care unit (ICU) and mechanical ventilation than non-pregnant women of childbearing age [2, 21]. Five percent of infected pregnant women were diagnosed with severe or critical forms of pneumonia requiring respiratory support [22]. In addition, in [23] the incidence of respiratory distress, fetal distress, miscarriage, preterm delivery and coagulopathy accompanied by liver dysfunction and even the mother’s death as a result of COVID-19 was observed in pregnant women.

Stress is the body’s response to adverse situations that most people encounter during their lifetime. Anxiety and stress affect 10.0 to 25.0% of pregnant women during pregnancy [3]. The COVID-19 pandemic was an atypical situation that generated stress factors that pregnant women had never encountered or only to a limited extent in non-pandemic times. During the pandemic, pregnant women were shown to experience significantly more pronounced symptoms of anxiety (57.0%) and depression (37.0%) compared to levels that commonly occur during pregnancy, as well as the levels experienced by other groups of the population during the current pandemic [3]. High levels of anxiety in pregnant women during the COVID-19 pandemic were also confirmed by [4–9]. Women were most often concerned about their health due to COVID-19 infection and the health of their fetus [3–5, 7, 9, 24]. Another significant stress factor was concerns regarding limited or inadequate healthcare (limitation of face-to-face prenatal visits, change of the birth plan, limited care due to overcrowded hospitals, unavailable equipment, different treatment in case of COVID-19 positivity) and isolation (inability of the support person to be present at the birth, restriction on hospital visits by family members) [3–5, 24]. Pregnant women were also worried about food shortages or job losses for close family members [4] and concerns about the health of elderly parents or grandparents infected with COVID-19 [5]. The three main groups of stress factors relating to health and healthcare during the COVID-19 pandemic will be discussed in more detail.

- *Fear of COVID-19 infection* - Naturally, fear of COVID-19 infection was one of the main stress factors [3–5, 7, 9, 24]. Even when complying with hygiene and quarantine measures, an increased risk of infection was demonstrated in public places [25, 26]. Thus, when visiting their doctor for regular prenatal check-ups, pregnant women were at risk of infection, especially when traveling by public transport [25] and in healthcare facilities (including individual departments, waiting rooms, hallways, elevators or toilets) [27–32]. In [27], a meta-analysis of nosocomial infections in people with COVID-19 was conducted. The proportion of nosocomial infections with the early onset of COVID-19 among confirmed cases was 44.0% (including medical staff, hospitalized patients and visitors). In [29], approximately 15.0% of patients hospitalized with COVID-19 were infected in hospital. Of these, 55.0% of cases were by patient-to-patient transmission through contact in the same bay. Another 14.0% of patients had contact with an infected person in the same department (using shared equipment or through the movement of staff). In the remaining cases, no obvious source of infection was found. The greatest fear of most pregnant women was being infected at healthcare facilities [4, 5, 24]. Some women were so afraid of giving birth in a hospital and staying on the ward with asymptomatic patients infected with COVID-19 that they explored the possibility of giving birth at home [24]. The case of a woman who decided to give birth at home without medical and midwife assistance due to her fear of COVID-19 infection in hospital is described in [33]. She did not even visit the hospital for subsequent postnatal care. In addition, in [4] there was a significant decrease in women who wanted to give birth at a maternity hospital, dropping from 96.4% before the pandemic to 87.7% during the pandemic. Some women even wanted to give birth prematurely by caesarean section for fear of infecting themselves or family members visiting the hospital [5].
- *Fear of transmitting the virus to the fetus* - Another significant factor was the fear among pregnant women that they could transmit the virus to the fetus and concerns regarding its health [3, 5, 7, 9]. The majority of women (83.2%) believed that the infection could be transmitted to the newborn, and a high proportion of women (84.6%) also believed that the infection could affect the health of the fetus if the mother was infected with the virus [34]. Transmission of the infection from mother to child takes place in the form of vertical transmission. Vertical transmission can be defined as transmission during pregnancy through the placenta in-utero, during delivery when the newborn comes into contact with the mother or after birth during breastfeeding [35]. Placental vertical transmission from mother to fetus has been demonstrated in COVID-19, provided transmission took place in the third trimester [20, 36–38]. The significant impact of the infection on the health of the newborn has not been demonstrated, but there is still insufficient information on whether the virus could have teratogenic effects if placental transmission were to occur in the first trimester [36]. In [35], children born to women with COVID-19 were shown to be at higher risk of admission to the neonatal ICU. In addition, pregnant women with COVID-19 were more likely to have a preterm delivery than women without this disease, and a high rate of cesarean sections was also shown in mothers with COVID-19 (in the range of 67.2–94.0%) [35].
- *Limited healthcare and isolation* - Insufficient or limited prenatal care was also frequently mentioned in connection with COVID-19 and stress factors [3, 4, 24]. According to a survey [3], 89.0% of pregnant women indicated changes in prenatal care during the pandemic, which was very stressful for them. These changes included the cancellation of doctor’s appointments (36.0%), the inability to bring a support person to the birth (90.0%), a change of birth plan (11.0%) or difficulties in accessing other healthcare (74.0%) such as psychological counseling or chiropractic care. The cancellation of doctor’s appointments as part of prenatal care was also noted by 25.8% of women in [4]. Pregnant women in [24] stated that they had to visit hospital departments located elsewhere as part of prenatal care during the pandemic and, in addition, they could not visit the hospital with their partner or have him/her at the birth. Twenty-eight percent of women reported that the frequency of regular check-ups was cut back and that some healthcare professionals were more concerned about COVID-19 than pregnancy. Fourteen percent of women mentioned they were concerned about how they would be treated by healthcare professionals if they were diagnosed with the virus. Most women were concerned about the availability of equipment (such as a birthing pool) and restrictions on their use [24]. There was also fear that the induction of labor would be canceled or delayed. Women complained that social isolation as part of postnatal care led to either a complete lack or reduction of support by family and friends [24, 39].

Remote monitoring could help reduce the impact of stress factors on pregnant women during the pandemic and reduce the risk of contracting COVID-19 or other viral diseases at healthcare facilities, in public spaces, as well as on public transport. Remote monitoring could become an alternative to regular doctor’s appointments, helping to protect pregnant women from infection, while monitoring their health from the comfort and safety of their home [40]. This form of remote healthcare could thus have a positive impact on both the mother’s physical and mental health.

## Past and present of fetal monitoring

This section provides the introduction to the evolution of the fetal monitoring, from the methods used in its early days until the state-of-the-art methods of today’s clinical practice.

### Evolution of fetal monitoring

**Fig. 1:**
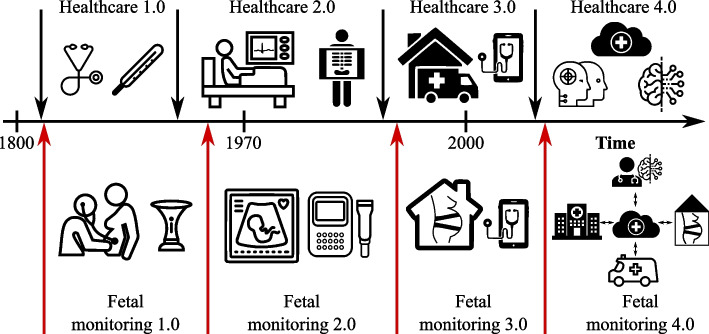
Timeline and major developments in healthcare and fetal monitoring

As in other sectors, fetal monitoring is changing and evolving, largely due to the rapid development of modern technologies. And so, like industrial development, we can talk about development and progress in healthcare (and consequently, fetal monitoring) marked by various milestones [41], as shown in Fig. 1. Industry 4.0 has transformed the manufacturing industry into a new paradigm. In a manner similar to manufacturing, healthcare delivery and fetal monitoring is at the dawn of a foundational change into a new era of smart and connected healthcare, referred to as Healthcare 4.0 [42]. Healthcare 4.0 is all about capturing enormous amounts of data and using it to make better healthcare management decisions and thus achieving significant gains in efficiency and cost control [43]. The evolution of healthcare according to Hathaliya et al. [44] is as follows:

1. *Healthcare 1.0* - the first healthcare revolution was characterized by major change in clinical practices and procedures, which became evidence-based and thus more efficient allowing the solution of major public health problems of that time (e.g., preventing infectious diseases by introducing vaccination). The patient’s records were paper-based and physically held by the physician.
2. *Healthcare 2.0* - new discoveries and inventions led to the introduction of new medical devices and methods in everyday practice. Technology became more prominent in healthcare, which enabled more accurate and early diagnosis and treatment.
3. *Healthcare 3.0* - as computers became smaller, they could be used in the form of home-monitoring and wearable devices, allowing continual telemonitoring of the patient and diagnosing their state of health more precisely. The Internet provided healthcare with continuous access and storage of data.
4. *Healthcare 4.0* - this collection of approaches facilitates the shift from hospital-centered to patient-centered care and making medicine more efficient and personalized. These approaches include, for example, the Internet of Things, artificial intelligence (AI), medical digital twins, robotics, and smart sensing. One of the steps needed in this phase is digitalization of the healthcare enterprise and services to provide enough input information to work with, and a large number of devices of varying types communicating with each other.

Similar concepts can be applied to fetal monitoring:

- *Fetal monitoring 1.0* – first monitoring based on maternal senses - information on fetal motility, contractions or other sudden changes. Later, the physician or the midwife could monitor the health of the fetus by means of intermittent auscultation, using either his/her ear or primitive tools such as the Pinard stethoscope - correlation between the fetal heart rate (fHR) and its well-being was established [45].
- *Fetal monitoring 2.0* – electronic fetal monitoring was introduced and replaced manual paper-based records. The fHR can be continually monitored with uterine contractions using the cardiotocograph [46].
- *Fetal monitoring 3.0* – successful introduction of a fetal telemedicine allowing remote fetal diagnosis and consultations with a specialist while reducing the costs and journey times [47]. This phase is thus associated with the development of wearable fetal monitoring devices that would allow the mother to monitor the health of the fetus at home [48].
- *Fetal monitoring 4.0* – the digitalization of healthcare is already underway, yet slowly compared to other industries. Four areas of digitalization will become increasingly familiar to healthcare professionals and patients in years to come:
  1. *Internet of medical things (IoMT)* – can allow patients to consolidate data, allowing clinicians to create a more precise picture of a patient’s health. Each fetal monitor can be considered an IoT device that is part of a vast network of the same devices [41]. Every device in such a network will then regularly connect to the internet to upload the measured results to remote servers, either for medical examination by an expert or simply for backup purposes [49].
  2. *Automation* – by incorporating automated equipment, smart monitoring, and decision-making services, the efficiency and accuracy of services for the patients may be increased. As a result, it will be possible to decrease the reliance on humans for mundane or repetitive tasks, speed up many of the services offered, and reduce the possibilities of errors negatively affecting the patients.
  3. *Artificial Intelligence* – using the power of predictive analytics to accelerate progress in fetal monitoring and diagnosis [50].
  4. *Cybersecurity* – in connection with the remote fetal monitoring project, the question of the management and storage of sensitive and key health data also arises. In general, data generated by measurement or during consultation with experts can be described as electronic medical records (EMR), i.e., critical, highly sensitive private information in healthcare [44]. Due to the sensitivity of this data, the question arises here on how to share the data efficiently, but safely. The answer to the question may be the use of blockchain technology [41].
  5. *Advanced Hybrid Technologies* – combining different technologies to enhance the quality of the sensed signal, such as fetal phonocardiography and electrocardiography [48].

### State-of-the-art fetal monitoring methods

The most commonly used method for electronic fetal monitoring in clinical practice today is cardiotocography, which monitors the fHR and uterine contractions [51]. However, there are other alternative methods based on different principles that seem to be much safer and more reliable options for remote home monitoring.

- *Fetal phonocardiography (fPCG)*﻿ – is a method based on sensing fetal heart sounds (fHS) through the mother’s abdominal wall. The advantage of this device is its simplicity given that cardiac activity can be detected using a medical stethoscope with a sensor in the form of a microphone or pressure sensor. The detected signal is converted into an electric signal, amplified, digitized and filtered [52]. This method is completely passive, low-cost and is a highly suitable alternative diagnostic tool for determining fHR. Due to its advantages, this method is suitable for home monitoring [53]. Among its main disadvantages is its high sensitivity to surrounding interference (noise) and the correct placement of the sensor. To obtain a good signal, the sensor must be placed as close as possible to the heart of the fetus, which can be difficult in earlier stages of pregnancy [52, 53].
- *Fetal echocardiography (fECHO)* ﻿– is a method based on Doppler ultrasound, which is used for detailed evaluation of fetal cardiac anatomy and the diagnosis of congenital fetal heart defects [54, 55]. The disadvantage of this method is the exposure of the mother and fetus to ultrasound radiation, and thus the inability to use this method for continuous monitoring. In addition, the evaluation of recordings relies on the physician’s knowledge and experience [55, 56].
- *Fetal magnetocardiography (fMCG)* ﻿– is a highly effective method based on measuring and analyzing the magnetic fields of the fetal heart using a superconducting quantum interference device (SQUID) [57]. The advantage of this method is the high quality of acquired signals, allowing accurate determination of fHR and accurate morphological analysis of the signal (ST analysis or QT interval analysis), which can be used for more precise determination of fetal hypoxia or to detect arrhythmias (e.g., supraventricular ectopy or atrial tachycardia) [58, 59]. However, this method is not available in hospitals very often due to its complexity (requires a shielded room and experienced specialist) and financial cost [57].
- *Fetal cardiotocography (CTG)* ﻿– this method is currently the most widely used fetal monitoring method in clinical practice. It uses a Doppler-based sensor to detect fHR and a pressure sensor to detect uterine contractions [51, 60]. Since both sensors are fastened to the mother’s abdomen with elastic bands, care must be taken to ensure optimal fastening. Insufficient or disproportionate pressure can distort measurements and cause the mother discomfort. The disadvantage of this method is that the sensor only senses heart beats and thus cannot provide more detailed information about fetal heart activity. In addition, the resulting fHR value is given by averaging instantaneous fHR values, which makes it difficult to assess short-term changes in fHR [61]. Evaluating CTG recordings requires an experienced professional. This method is also sensitive to the correct placement of the sensor and motion artifacts [62]. Sensitivity is reduced in women with a high body mass index (BMI) [63]. Another disadvantage is the exposure of the mother and fetus to ultrasound radiation and the mother’s inability to move freely during monitoring [61].
- *Fetal electrocardiography (fECG)* ﻿– fECG is used to record the electrical activity of the fetal heart. There is an invasive form of this method (I-fECG), in which fetal heart activity is sensed using a scalp electrode placed directly on the fetus’s head and a non-invasive form (NI-fECG), in which heart activity is recorded from the pregnant woman’s abdominal region [64]. Although the invasive approach provides a very good signal, it can only be performed during labor and there is also the risk of infection [65]. The non-invasive option is safe compared to the invasive version and allows monitoring during pregnancy and labor. Compared to CTG, fECG can provide signal morphology in addition to fHR, which gives more precise information about the health of the fetus (it can more accurately detect fetal hypoxia [61], and the morphology of fECG signals is also expected to contain much more information about heart defects compared to conventional sonographic methods [64]). What’s more, the mother and fetus are not exposed to ultrasound radiation, which allows continuous monitoring and monitoring as part of home care. The method is also suitable for women with a higher BMI [66]. For all the above reasons, fECG could become the alternative to CTG in clinical practice. The main disadvantage of this method is the high degree of interference caused by the maternal electrocardiographic signal (mECG), which is detected together with the useful fECG signal [61].

A comparison of fetal monitoring methods is given in Table 1. The table summarizes the types of sensors these methods use and the stage of pregnancy from which the method can be applied. It further specifies whether it is possible to determine the fHR or perform a morphological analysis of the acquired signal using the method. The table also contains information on whether it is a non-invasive or invasive method, and the last column defines whether the mother can move freely when using the device.

**Table 1:** Comparison of fetal monitoring methods

Monitoring method	Senzor type	Week of pregnancy	fHR	Morphological analysis	Non-invasive	Mobility
fPCG	Microphones or pressure transducers	$\ge$ 20	Yes	No	Yes	No
fECHO	Ultrasound transducer	$\ge$ 18	Yes	No	Yes	No
fMCG	Superconducting quantum interference device senzor	$\ge$ 14	Yes	Yes	Yes	No
CTG	Ultrasound transducer and pressure transducer	$\ge$ 28	Yes	No	Yes	No
I-fECG	Scalp spiral electrode	Only during labor	Yes	Yes	No	No
NI-fECG	Standard ECG electrodes	$\ge$ 20	Yes	Yes	Yes	Yes

The NI-fECG is often praised to be one of the most promising methods for continuous monitoring, which is in consistent with the findings summarized in Table 1. Although the extraction of the fECG signal using the implemented algorithm is accurate, there are cases (e.g., unsuitable fetal position in the mother’s abdomen during measurement) when the fetal component in aECG signals becomes very low due to the maternal component and the suppression of mQRS complexes is challenging [67]. In these cases, the algorithm cannot reliably completely suppress the residual maternal component. These residues can be detected as false positive fQRS complexes and the value of the resulting fHR can be distorted [68, 69]. To avoid such inaccurate measurement, it would be thus beneficial to combine it with another monitoring method, such as fPCG, which is the second most promising method for continuous fetal monitoring.

The combination of these two methods has achieved promising results in case of the preliminary study reported by Gobillot et al. [70]. Simultaneous measurement of fECG and fPCG yielded an fHR curve that highly correlated with the fHR curve obtained using the reference CTG. Electrical signals were acquired from both the mother’s chest and abdomen, and acoustic signals were measured using microphones. However, testing has only been performed on 9 recordings obtained from 7 women between the 24$^\mathrm {th}$ and 39$^\mathrm {th}$ week of pregnancy, and much more research is needed.

The combination of fECG and fPCG has also been implemented in some of the commercially available systems, such as *Invu System* [48], which is a wearable, self-administered, fixed location device in the form of a belt with 12 sensors (8 passive electrical and 4 acoustic sensors-microphones). Measured fECG and fPCG signals were independently processed and analyzed. The results from electrical and acoustic signals were subsequently merged and the mHR and fHR were determined. The device was tested on 147 women with singleton pregnancies from the 32$^\mathrm {nd}$ week of pregnancy. Although the device is primarily designed for remote home monitoring, in this first phase, measurements were conducted by research staff in a medical setting. Both mHR and fHR values were compared with values obtained using concurrent CTG measurements. The results showed a high correlation between the *Invu System* and CTG.

By implementing a combination of fECG and fPCG methods, it would be possible to obtain more precise clinical information about the health of the fetus, or to obtain such information in cases where it cannot be obtained with fECG at all (e.g., between the 28$^\mathrm {th}$ and 32$^\mathrm {nd}$ week of pregnancy, when the fetus is covered with a thick electrically non-conductive layer of *vernix caseosa* and fECG is virtually impossible to measure) [64]. In addition, fPCG can provide additional information about the health of the fetus (e.g., information on heart murmurs) that cannot be detected by fECC [52].

### Available fetal monitoring devices

Researchers and scientists have devoted extensive time and effort to research in the field of NI-fECG. Most studies [71–73] only focus on testing algorithms for filtering fECG; only a few authors deal with the comprehensive design of a prototype for detecting, processing and transmitting fECG in the context of a remote home monitoring system. These include Signorini et al. [74], who designed the wearable *Telefetalcare System* which, according to the accuracy parameter (ACC), achieved an overall accuracy of 98.5% in the detection of mQRS complexes and 91.3% in the detection of fQRS complexes. Signals and the accuracy of detection were evaluated by a specialist (physician). The *Prototype for Diagnosing Fetal Arrhythmia* was designed by Corona-Figueroa et al. [75]. In addition to determining mHR and fHR, the device can also detect fetal arrhythmias (bradycardia, sinus bradycardia, tachycardia, sustained supraventricular tachycardia, atrial flutter, accelerations, decelerations and prolonged decelerations). When tested on synthetic signals, it achieved 98.6% accuracy in determining fHR and 88.9% in detecting arrhythmia. The *Fetal ECG Monitoring System Based on the Android Smartphone* was designed by Yuan et al. [76] and the Savvy Sensor was designed by Rashkovset al. [77]. Examples of prototypes for NI-fECG monitoring are compared in Table 2.

**Fig. 2:**
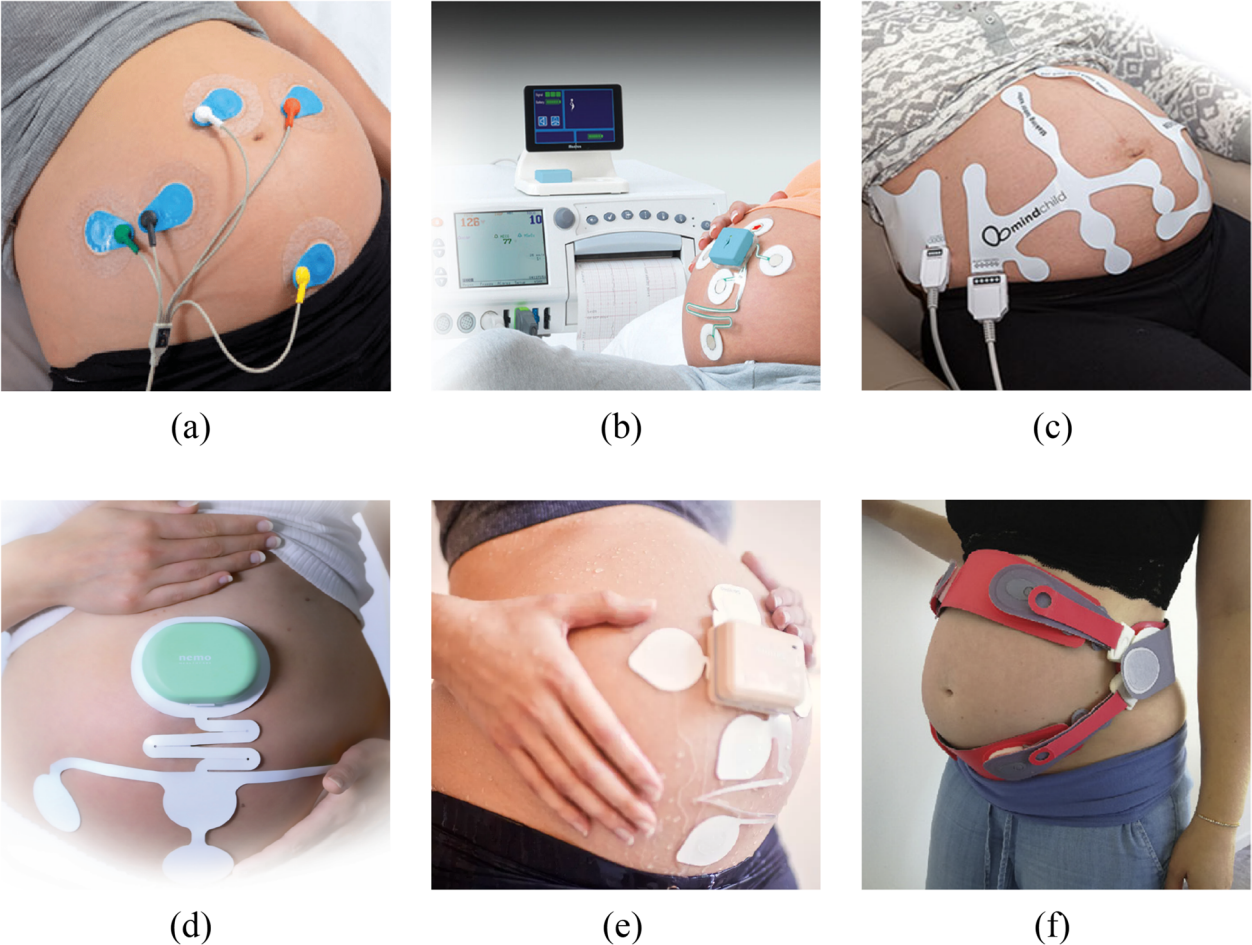
Examples of certified commercial fetal monitoring devices, **a** Monica AN24, **b** Novii Wireless Patch System [80], **c** MERIDIAN M110 Fetal Monitoring System [81], **d** Nemo Fetal Monitoring System [83], **e** Avalon Beltless Fetal Monitoring Solution [85] and **f** Invu System [48]

In recent years, the manufacturers of commercial fetal monitoring devices have also begun to notice the advantages of NI-fECG listed above. However, their number is still limited, as there are only six manufacturers that have obtained US food and drug administration (FDA) or CE certification for their devices, which can therefore be used in clinical practice in Europe. The first is Monica Healthcare Ltd. (Nottingham, UK), which manufactures the Monica AN24 [78, 79]. The second is GE Healthcare (Chicago, IL, USA), which manufactures the *Novii Wireless Patch System* [80]. The third is MindChild Medical, Inc. (North Andover, MA, USA), which manufactures the *MERIDIAN M110 Fetal Monitoring System* [78, 81, 82]. Another is Nemo Healthcare (Veldhoven, the Netherlands), which manufactures the *Nemo Fetal Monitoring System* [83, 84] and Koninklijke Philips N.V. (Amsterdam, The Netherlands), which manufactures the *Avalon Beltless Fetal Monitoring Solution* [85]. The last manufacturer is NUVO Inc. (Erie, PA, USA), which manufactures the wireless *Invu System* [48], which is the only device from those listed focused on home monitoring in different stages of pregnancy rather than monitoring during the labour. Examples of commercial NI-fECG monitoring devices are shown in Fig. 2 and compared in Table 2. The table summarizes the types of sensors these methods use and the stage of pregnancy from which the method can be applied. It further specifies whether it is possible to determine the fHR, the maternal heart rate (mHR) or to perform a morphological analysis of the acquired signal using the method. The last column shows the certification of the device.

## Future prospects in pregnancy monitoring

The previous sections introduced the state of the state-of-the-art methods and technology and revealed the need for the development in the field of fetal monitoring catalyzed by the surge of the global pandemic. This section aims to introduce some of the possible future directions of the research, summarized by the newly introduced naming: Fetal Monitoring 4.0 (see 4.1), as well as the areas where the AI can be implemented.

**Table 2:** Comparison of commercially available and prototype fetal monitoring devices

Monitoring device	Senzor type	Week of pregnancy	fHR, mHR	Uterine activity	Morphological analysis	Certification
Monica AN24	5 standard ECG electrodes	$\ge$ 20	Yes	Yes	No	FDA, CE
Novii Wireless Patch System	Patch system with 5 incorporated electrodes	$\ge$ 36	Yes	Yes	No	FDA, CE
MERIDIAN M110 Fetal Monitoring System	Patch system with 28 incorporated electrodes	$\ge$ 36	Yes	Yes	No	FDA
Nemo Fetal Monitoring System	Patch system with 6 incorporated electrodes	$\ge$ 21	Yes	Yes	No	CE
Avalon Beltless Fetal Monitoring Solution	Patch system with incorporated electrodes	–	Yes	Yes	No	FDA, CE
Invu System	Belt system with 8 electrical senzores and 4 microphones	$\ge$ 32	Yes	No	No	FDA
Telefetalcare System	Bodysuit with 9 ECG textile electrodes	$\ge$ 37	Yes	No	No	–
Prototype for Diagnosing Fetal Arrhythmia	2 sets of 3 ECG electrodes	$\ge$ 32	Yes	No	No	–
Fetal ECG Monitoring System Based on the Android Smartphone	5 standard ECG electrodes	–	Yes	No	No	–
Savvy Sensor	2 electrodes in a plastic housing	$\ge$ 20	Yes	No	No	–

### Concept of fetal monitoring 4.0

**Fig. 3:**
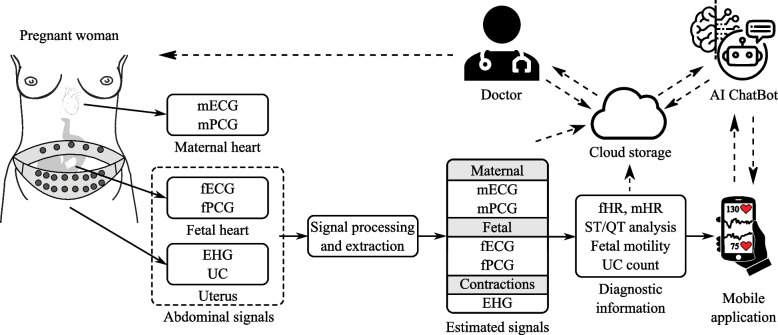
Schematic diagram of the concept of Fetal Monitoring 4.0 based on NI-fECG

Herein, we suggest a concept of Fetal Monitoring 4.0 based which aims to balance out the drawbacks of the state-of-the-art methods summarized above and follow the current trends towards the e-Health and Healthcare 4.0. The concept is illustrated by means of the schematic diagram in Fig. 3. The whole concept consists of the following units:

1. *Fetal monitoring system* – includes a fetal measuring pad system (herein referred to as measuring belt but the technology may vary) consisting of multiple measuring electrodes/probes based on the principle used. This system acquires the abdominal signals (e.g. ECG or PCG) from the maternal skin surface. These signals include both a maternal and fetal components and need to be further processed in order to obtain the fetal signal and associated clinical features.
  - *Electrode/sensor placement* – the measuring probe’s placement is a challenging task in fetal monitoring. For most of the available methods, there is no standard such as in the case of monitoring in adults. In the case of NI-fECG, the optimal electrode selection varies based on different factors, such as the stage of pregnancy or fetal position [61]. Therefore, it is advantageous to create a general electrode placement covering a reasonable area of the maternal abdomen [86]. However, we must keep in mind that the number of measuring probes should not be too high due to significant energy demands for continual monitoring. The most feasible solution is thus a measuring device in the form of an adjustable wearable belt with incorporated measurement probes. However, there were also been attempts for the measurement of the signals using smart textiles [87].
  - *Materials used* – besides demands on the quality of the signal being sensed, the wearable system should also comply with requirements for the patient’s safety and comfort. This is associated with the materials used. Both the wearable belt and measuring probes should be made from hypoallergenic, biocompatible materials. Among the preferable materials are thus antimicrobial nanotextiles providing protection against the formation of microorganisms during moisture or sweating. The materials should also be flexible (to adjust to the varying shape and size of the pregnant woman’s abdomen) and durable (for daily use). The trend is in reducing the size of sensors.

As summarized in State-of-the-art fetal monitoring methods section, most prototypes for NI-fECG monitoring use disposable patch systems. This approach, however, is not suitable for continuous home monitoring in commercial systems. As suggested by adult ECG monitoring, the most suitable sensors for this task are wearable, incorporated into textiles or other suitable materials [88, 89].

2 *Signal analysis tool* – this tool enables processing of input abdominal signals in order to the fetal component and extract the clinical features, such as fHR. The tool should include functions allowing following steps:
  - *Preprocessing and signal extraction* – because acquired abdominal signals contain the maternal component and other interference in addition to the useful fetal component, this interference must first be eliminated. The first step is pre-processing, which supresses most of the unwanted signals in a given range, which varies according to the signal being measured. For example, in NI-fECG, the pre-processing with a bandpass finite impulse response filter with cut-off frequencies of 3 and 150 Hz and a filter order of 500 is sufficient to eliminate conventional interference (e.g., isoelectric line fluctuations, respiratory artifacts or motion artifacts) [68]. A more complex task arises in eliminating unwanted signals, especially the maternal component, as the measured signals overlap in time and frequency. In addition, the amplitude of fetal component tends to be several times lower than the maternal one, and so, the use of advanced extraction methods is unavoidable [67]. A number of algorithms have been successfully tested for both the fPCG and fECG extraction in the past, including independent component analysis (ICA) [90], principal component analysis (PCA) [91], empirical mode decomposition-based (EMD) [92] algorithms, wavelet transform (WT) [93], adaptive neuro-fuzzy inference system (ANFIS) [94] and adaptive algorithms such as least mean squares (LMS) [93], recursive least squares (RLS) [95] or fast transversal filter (FTF) [96]. When selecting an algorithm, in case of fECG extraction, it is necessary to ensure sufficient suppression of the maternal component so that maternal R peaks are not detected and the fHR can be accurately determined [67, 68]. Also, the resulting fECG signal should not be distorted in order to enable a morphological analysis (e.g., ST analysis or QT interval analysis), which provides additional valuable information on fetal health [97].
  - *Fetal heart rate determination* – fetal heart rate is one of the most commonly used parameters in fetal monitoring. It is associated with the amount of beats occuring in a minute. The fetal beats can be obtained from the measured signals, where significant waves/peaks are detected. In NI-fECG, the R peaks are detected in the extracted fECG signal with the suppressed maternal component [69], whereas in fPCG, the significant feature for the detection is the S1 sound [98]. Following the reliable detection of fetal R peaks/S1 sounds, the current fHR can be determined or the fHR trace plotted over time.
  - *Maternal heart rate determination* – since the maternal component is dominant in the abdominally sensed signal, it is much easier to detect maternal R peaks/S1 sounds than the fetal ones. Using these peaks, the mHR can be determined and displayed along with information about the fetus.
  - *Uterine contraction detection* – The frequency of uterine contractions, their length and intensity indicate the condition of the uterus, which will help the obstetrician identify the progress of labor and whether hospitalization is already needed. In the NI-fECG method, uterine contractions can be sensed using an electrohysterogram (EHG), which is the signal relating to action potentials generated by the uterus that propagate to the maternal abdomen and can thus be sensed along with the aECG signal [61]. As for the fPCG method, the information about uterine contractions can also be obtained as proposed in [99]. The abdominally sensed acoustic signals are modulated by the uterine contractions. The measured abdominal PCG signals altered in this way thus have detectable low-frequency changes in their morphology and this information can be extracted by basic filtering methods.
3 *Mobile/PC application* – for efficient remote monitoring, an application should be created for the needs of both the clinicians pregnant women to obtain information about the health of the fetus. For pregnant women, such application should have a user-friendly environment providing simple information about the health, further information should be only provided by the physicians. In contrast, the interface for clinicians should be designed to provide further analysis of the measured signals. The application should include, for example, automatic *The International Federation of Gynecology and Obstetrics* (FIGO) guidelines classification [100], fHR in time, fHRV analysis or morphological analysis of the NI-fECG signal, such as ST analysis.
4 *Communication network* – data is stored and can be viewed or shared with other specialists. This way, fetal monitoring could become part of the IoMT.
5 *Cloud storage* – allows a large amount of obtained health data about the mother and fetus to be stored. The main benefit is its flexibility, fast access and easy updates of medical records. The patient’s entire medical history can be found in one place, which can be accessed by other members of the medical team, who can act on the basis of current information about the patient’s health.

### Implementation of artificial intelligence

Medical technologies equipped with AI are rapidly evolving into clinical practice. In fetal monitoring, however, the progress is slower than in other field particularly due to the fact that there is no high-quality dataset with large amount of real physiological or pathological recordings available for the fECG method. When implementing the Fetal monitoring 4.0 concept, the data could be easily collected, pre-processed, and stored, which would enable implementing AI-based solutions in the key application areas:

1. *Algorithm Optimization* – although fECG signal extraction using the proposed concept achieves promising results in most cases, there is still room for improvement. One of the main factors affecting the quality of resulting fECG signals is the optimal setting of extraction algorithm parameters. If the parameters affecting the quality of filtration are set correctly, the resulting fECG signal is good and can be used to extract the maximum amount of clinical information [61, 69]. However, biological signals, including fECG signals, and the noise associated with fECG signals, are nonlinear, dynamic and variable, and the optimal setting of algorithm parameters is therefore different for each signal. When processing each fECG signal, it is necessary to individually find and set a different combination of extraction algorithm parameters. This process is called algorithm optimization. The most commonly used optimization approaches are grid search and random search [101]. However, these approaches are very time consuming (especially when setting parameters acquiring continuous values) and do not guarantee finding the global optimum. The use of meta-heuristic methods, such as particle swarm optimization (PSO) [102], differential evolution (DE) [103] or moth flame optimization (MFO) [104], seems more appropriate. These approaches excel in the ability to find the global optimum and avoid getting stuck in the local optimum. In addition, they can find the global optimum faster and more efficiently, which is an important criterion if the algorithm is to be implemented in a real-time device. Unfortunately, optimization in the field of fECG extraction has been addressed by a small number of authors so far, and further research in this area is needed. These authors include, for example, Nasiri et al. [105] or Elmansouri et al. [106] who tuned the ANFIS algorithm using PSO. Using the combined algorithm, the authors achieved better fECG extraction and the results were obtained faster compared to conventional ANFIS. PSO also proved effective in the optimization of extended Kalman smoother according to Panigraha et al. [107]. The DE method by Kockanat et al. [108] was implemented for the purposes of optimizing the parameters of the adaptive algorithm. The DE algorithm optimized the coefficient vector of the adaptive filter. Based on a visual comparison of extracted signals, residual mQRS complexes were completely suppressed using the combined algorithm. Whereas when using a conventional LMS filter, residual mQRS complexes were visible. Panigrahy et al. [109] also achieved highly accurate extractions (accuracy 90.7%) without the need for the initialization of parameters by the user when combining DE with extended Kalman smoother and ANFIS. The relatively new MFO algorithm introduced in 2015 also proved to be suitable in the field of fECG extraction. It was tested by Jibia et al. [110] in combination with the adaptive LMS algorithm. Extraction using this combined method proved promising, and the MFO also excelled in its simple implementation and flexibility.
2. *Automated Classification* – Although the concept of Fetal Monitoring 4.0 allows basic automatic classification of fetal health, a larger number of features should be used in the future for a more comprehensive analysis, including both the morphological properties of the fECG curve and fHR properties in terms of time and frequency parameters, all in relation to uterine contraction features. The analysis and subsequent classification of a large number of extracted elements can be difficult for conventional algorithms. However, algorithms based on AI and machine learning, which excel in solving complex tasks and processing large amounts of data, could find their application in the automatic classification of extracted signals or state of fetal health. Algorithms based on AI, such as artificial neural networks [50], support vector machines [111] or random forest [112] have been used to classify CTG recordings in the past to detect fetal hypoxia. The determination of fetal hypoxia by a physician visually evaluating fHR traces and uterine contractions often led to less than satisfactory results and was influenced by the subjective evaluation and experience of the physician. For these reasons, research focused on testing automatic AI-based algorithms for the interpretation of CTG recordings. The principle is based on the extraction of elements from both the fHR trace and uterine contraction curve (e.g., baseline fHR, number of prolonged decelerations per second or uterine contractions per second) and classifying the recording into one of three classes: *normal*, *suspicious* or *pathological* [111, 113]. Since it is possible to estimate a quality fHR curve from the fECG signal and obtain information on uterine contractions, such as in the case of CTG, the use of AI to classify fECG is one of the main directions that further research should take. In addition, unlike CTG, it is possible to extract morphological elements (e.g., ST segment or QT interval) from the fECG signal, which can help provide much more precise information on fetal health, especially in connection with hypoxia. It would also be highly beneficial to implement an algorithm for classifying uterine contractions in the future. The algorithm would be able to identify whether these are Braxton-Hick’s contractions or truelabor contractions based on information on frequency, duration, and intensity. The pregnant woman would then be sure whether she needs to go to the hospital or can stay at home, which would allow the mother to spend the maximum amount of time in her home environment and the time spent in the hospital with the risk of contracting COVID-19 (or any other viral disease) would be minimized.
3. *Automated Question-Answering Systems* – various health complications can occur during pregnancy, which may or may not be serious. Despite the fact that a large amount of information can be found on the Internet today, some situations may be specific, especially during pregnancy, and this information may not always be easily traceable, which can lead to stress in pregnant women. In many cases, the lack of information and stress lead to higher number of in-person prenatal care visits, which are often unnecessary and, in the group of low-risk patients, may lead to higher rates of pregnancy interventions without improvement in neonatal outcomes [114]. Several authors proposed so-called chatbot services for the use in healthcare [115]. These systems are often based on AI [116, 117] and unconstrained natural language input capabilities [118]. The questions that are given by the user are answered by the system which creates its responses by querying a structured database including given information [119]. Among the common purposes of the conversation agents are data collection, clinical interviews, and clinical decision support purposes [115]. They have been used in various healthcare domains, such as mental health [120, 121], patient monitoring (pain [122], hypertension [123, 124], diabetes [125–127]). Most recently, chatbots also found the utilization in countering the COVID-19 pandemic [128–131], namely in disseminating important health information; self-triage and risk assessment; monitoring exposure; tracking symptoms and health outcome; and fighting misinformation and fake news [132]. In fetal monitoring, the chatbots could be particularly useful to simulate the healthcare professional in home monitoring between the prenatal check-ups [133, 134]. If the pregnant woman does not find an answer using this system, she could be then asked to contact her physician. Such a system could help pregnant women deal with health complications according to professional medical procedures and recommendations without unnecessary stress to the patient while decreasing the number of unnecessary in-person prenatal care visits, especially in the low-risk patients, similarly as in [135]. The approach would be also helpful for proper monitoring in rural areas facing lack of appropriate alerting mechanism in case of abnormal gestation evolution [136].

## Discussion of possible obstacles

This section summarizes the challenges that should be addressed in the future and outlines the directions that the development of the concept of NI-fECG monitoring should take. Algorithms based on AI should be implemented to fulfill the other pillars of Healthcare 4.0. The use of methods based on AI could be beneficial, for example, in the optimization of extraction algorithms or the classification of extracted signals, and thereby the state of the fetus’s health. However, an emphasis should be placed on the accuracy and the interpretation of the results to avoid false alarms which could only cause stress to the pregnant woman.

Moreover, as measured maternal and fetal data is highly sensitive private information, emphasis must be placed on its effective and safe management. In general, cloud storage is considered highly secure, but centralized storage. From an EMR management perspective, this can be a significant problem, as there may be situations where a system based on one centralized server becomes unavailable (for example, in case of cyberattacks on hospitals, the number of which is has recently increased [137]) and patient information does not reach the doctor in time. A situation could also arise where the data is modified or deleted altogether. The solution to these limitations could be the implementation of blockchain technology.

## Conclusion

The concept of Fetal Monitoring 4.0 for remote monitoring of fetal health from the comfort of home is a very promising tool for reducing the risk of contracting COVID-19 or other infectious diseases by pregnant women. The device is based on NI-fECG technology, which, unlike conventionally used CTG, does not emit ultrasound radiation and can be used for continuous measurements during pregnancy and labor. In addition, it is possible to obtain more accurate information about the health of the fetus from NI-fECG than in the case of CTG, and it is also possible to measure fECG in women with a higher BMI. The device enables the measurement of an aECG signal from the mother’s abdomen using an adjustable wearable belt with incorporated electrodes, the extraction of fECG and classification of the health of the fetus. This data can be viewed using a user-friendly mobile application, shared with a doctor and stored in cloud storage. To make this device even more reliable, future research should focus on incorporating other pillars of Healthcare 4.0 such as the implementation of artificial intelligence to optimize the extraction algorithm, for more comprehensive automated classification of fetal health, or automated question-answering systems. This monitoring method should be combined with other non-invasive fetal monitoring methods, such as fPCG, to provide more precise health information and to overcome the limitations of NI-fECG. To increase security in the management and storage of sensitive data on the health of the mother and fetus, technology based on decentralized blockchain technology should be incorporated in the future.

## Data Availability

There are no data to make available.

## Abbreviations

ACC: accuracy
ANFIS: adaptive neuro-fuzzy inference system
AI: artificial intelligence
BMI: body mass index
CTG: cardiotocography
DE: differential evolution
RLS: recursive least squares
EHG: electrohysterogram
EMR: electronic medical records
EMD: empirical mode decomposition
FTF: fast transversal filter
fECHO: fetal echocardiography
fECG: fetal electrocardiography
fHR: fetal heart rate
fHS: fetal heart sounds
FIGO: International Federation of Gynecology and Obstetrics
fMCG: fetal magnetocardiography
fPCG: fetal phonocardiography
FDA: food and drug administration
ICA: independent component analysis
ICU: intensive care unit
IoMT: internet of medical things
NI-fECG: invasive fetal electrocardiography
mECG: maternal electrocardiographic signal
mHR: maternal heart rate
MFO: moth flame optimization
NI-fECG: non-invasive fetal electrocardiography
PSO: particle swarm optimization
PCA: principal component analysis
SARS-CoV-2: severe acute respiratory syndrome coronavirus 2
SQUID: superconducting quantum interference device
WHO: World Health Organization

## References

[1] Goyder C (2020). COVID-19: Crystallising the Importance of Patient Safety. Br J Gen Pract..

[2] World Health Organization. Coronavirus disease (COVID-19): pregnancy and childbirth: World Health Organization; 2020. Available: https://www.who.int/. Accessed 1 Jan 2022.

[3] Lebel C, MacKinnon A, Bagshawe M, Tomfohr-Madsen L, Giesbrecht G (2020). Elevated Depression and Anxiety Symptoms among Pregnant Individuals during the COVID-19 Pandemic. J Affect Disord..

[4] Moyer CA, Compton SD, Kaselitz E, Muzik M (2020). Pregnancy-Related Anxiety during COVID-19: A Nationwide Survey of 2740 Pregnant Women. Arch Womens Mental Health..

[5] Salehi L, Rahimzadeh M, Molaei E, Zaheri H, Esmaelzadeh-Saeieh S. The Relationship among Fear and Anxiety of COVID-19, Pregnancy Experience, and Mental Health Disorder in Pregnant Women: A Structural Equation Model. Brain Behav. 2020;10(11). 10.1002/brb3.1835. 10.1002/brb3.1835 PMC7536966 32969190

[6] Abdoli A, Falahi S, Kenarkoohi A, Shams M, Mir H, Jahromi MAM (2020). The COVID-19 Pandemic, Psychological Stress during Pregnancy, and Risk of Neurodevelopmental Disorders in Offspring: A Neglected Consequence. J Psychosom Obstet Gynecol..

[7] Mortazavi F, Mehrabadi M, KiaeeTabar R (2021). Pregnant Women’s Well-Being and Worry during the COVID-19 Pandemic: A Cross-Sectional Study. BMC Pregnancy Childbirth..

[8] Asai K, Wakashima K, Toda S, Koiwa K (2021). Fear of Novel Coronavirus Disease (COVID-19) among Pregnant and Infertile Women in Japan. J Affect Disord Rep..

[9] Nausheen S, Bhamani S, Makhdoom A, Sheikh L (2020). Fear of COVID-19 among Pregnant Women in Pakistan: A Cross-Sectional Study. Int J Community Med Public Health..

[10] Cardwell MS (2013). Stress: Pregnancy Considerations. Obstet Gynecol Surv..

[11] Coussons-Read ME (2013). Effects of Prenatal Stress on Pregnancy and Human Development: Mechanisms and Pathways. Obstet Med..

[12] Yu Y, Zhang S, Wang G, Hong X, Mallow EB, Walker SO (2013). The Combined Association of Psychosocial Stress and Chronic Hypertension with Preeclampsia. Am J Obstet Gynecol..

[13] Ben-Ami I, Maymon R, Svirsky R, Cuckle H, Jauniaux E (2013). Down Syndrome Screening in Assisted Conception Twins: An Iatrogenic Medical Challenge. Obstet Gynecol Surv..

[14] Dunkel Schetter C, Tanner L (2012). Anxiety, Depression and Stress in Pregnancy: Implications for Mothers, Children, Research, and Practice. Curr Opin Psychiatry..

[15] de Weerth C, Buitelaar JK (2005). Physiological Stress Reactivity in Human Pregnancy–a Review. Neurosci Biobehav Rev..

[16] Dashraath P, Wong JLJ, Lim MXK, Lim LM, Li S, Biswas A (2020). Coronavirus Disease 2019 (COVID-19) Pandemic and Pregnancy. Am J Obstet Gynecol..

[17] Chiu NC, Chi H, Tai YL, Peng CC, Tseng CY, Chen CC (2020). Impact of Wearing Masks, Hand Hygiene, and Social Distancing on Influenza, Enterovirus, and All-Cause Pneumonia During the Coronavirus Pandemic: Retrospective National Epidemiological Surveillance Study. J Med Internet Res..

[18] Alimohamadi Y, Holakouie-Naieni K, Sepandi M, Taghdir M (2020). Effect of Social Distancing on COVID-19 Incidence and Mortality in Iran Since February 20 to May 13, 2020: An Interrupted Time Series Analysis. Risk Manag Healthc Policy..

[19] Sun B, Yeh J (2020). Mild and Asymptomatic Covid-19 Infections: Implications for Maternal, Fetal, and Reproductive Health. Front Reprod Health..

[20] Yee J, Kim W, Han JM, Yoon HY, Lee N, Lee KE (2020). Clinical Manifestations and Perinatal Outcomes of Pregnant Women with COVID-19: A Systematic Review and Meta-Analysis. Scientific Reports..

[21] Wastnedge EAN, Reynolds RM, van Boeckel SR, Stock SJ, Denison FC, Maybin JA (2021). Pregnancy and COVID-19. Physiol Rev..

[22] Adhikari EH, Moreno W, Zofkie AC, MacDonald L, McIntire DD, Collins RRJ (2020). Pregnancy Outcomes Among Women With and Without Severe Acute Respiratory Syndrome Coronavirus 2 Infection. JAMA Netw Open..

[23] Panahi L, Amiri M, Pouy S. Risks of Novel Coronavirus Disease (COVID-19) in Pregnancy; a Narrative Review. Arch Acad Emerg Med. 2020;8(1). 10.22037/aaem.v8i1.595. PMC7092922 32232217

[24] Karavadra B, Stockl A, Prosser-Snelling E, Simpson P, Morris E (2020). Women’s Perceptions of COVID-19 and Their Healthcare Experiences: A Qualitative Thematic Analysis of a National Survey of Pregnant Women in the United Kingdom. BMC Pregnancy and Childbirth..

[25] Tirachini A, Cats O. COVID-19 and Public Transportation: Current Assessment, Prospects, and Research Needs. J Public Transp. 2020;22(1). 10.5038/2375-0901.22.1.1. 10.5038/2375-0901.22.1.1 PMC9468467 36118518

[26] Ahmad T, Khan M, Haroon, Musa TH, Nasir S, Hui J, et al. COVID-19: Zoonotic Aspects. Travel Med Infect Dis. 2020;36:101607. 10.1016/j.tmaid.2020.101607. 10.1016/j.tmaid.2020.101607 PMC7128549 32112857

[27] on behalf of COVID-19 Evidence and Recommendations Working Group, Zhou Q, Gao Y, Wang X, Liu R, Du P, et al. Nosocomial Infections among Patients with COVID-19, SARS and MERS: A Rapid Review and Meta-Analysis. Annals of Translational Medicine. 2020;8(10):629. 10.21037/atm-20-3324. 10.21037/atm-20-3324 PMC7290630 32566566

[28] Ji H, Liu L, Huang T, Zhu Y (2020). Nosocomial Infections in Psychiatric Hospitals during the COVID-19 Outbreak. Eur J Psychiatr..

[29] Rickman HM, Rampling T, Shaw K, Martinez-Garcia G, Hail L, Coen P (2021). Nosocomial Transmission of Coronavirus Disease 2019: A Retrospective Study of 66 Hospital-Acquired Cases in a London Teaching Hospital. Clinical Infectious Diseases..

[30] Wang X, Zhou Q, He Y, Liu L, Ma X, Wei X (2020). Nosocomial Outbreak of COVID-19 Pneumonia in Wuhan, China. Eur Respir J..

[31] Klompas M (2020). Coronavirus Disease 2019 (COVID-19): Protecting Hospitals From the Invisible. Ann Intern Med..

[32] Van Praet JT, Claeys B, Coene AS, Floré K, Reynders M (2020). Prevention of Nosocomial COVID-19: Another Challenge of the Pandemic. Infect Control Hosp Epidemiol..

[33] Nosratabadi M, Sarabi N, Masoudiyekta L. A Case Report of Vaginal Delivery at Home Due to Fear of Covid-19. Iran J Psychiatry. 2020. 10.18502/ijps.v15i4.4306. 10.18502/ijps.v15i4.4306 PMC7610078 33240387

[34] Hossain N, Samuel M, Sandeep R, Imtiaz S, Zaheer S. Perceptions, Generalized Anxiety and Fears of Pregnant Women about Corona Virus Infection in the Heart of Pandemic. 2020. 10.21203/rs.3.rs-32235/v1.

[35] Madjunkov M, Dviri M, Librach C (2020). A Comprehensive Review of the Impact of COVID-19 on Human Reproductive Biology, Assisted Reproduction Care and Pregnancy: A Canadian Perspective. J Ovarian Res..

[36] Kotlyar AM, Grechukhina O, Chen A, Popkhadze S, Grimshaw A, Tal O (2021). Vertical Transmission of Coronavirus Disease 2019: A Systematic Review and Meta-Analysis. Am J Obstet Gynecol..

[37] Vivanti AJ, Vauloup-Fellous C, Prevot S, Zupan V, Suffee C, Do Cao J (2020). Transplacental Transmission of SARS-CoV-2 Infection. Nat Commun..

[38] Ashraf MA, Keshavarz P, Hosseinpour P, Erfani A, Roshanshad A, Pourdast A (2020). Coronavirus Disease 2019 (COVID-19): A Systematic Review of Pregnancy and the Possibility of Vertical Transmission. J Reprod Infertility..

[39] Nowacka U, Kozlowski S, Januszewski M, Sierdzinski J, Jakimiuk A, Issat T (2021). COVID-19 Pandemic-Related Anxiety in Pregnant Women. Int J Environ Res Public Health..

[40] Wu D, Fang D, Wang R, Deng D, Liao S (2021). Management of Pregnancy during the COVID-19 Pandemic. Glob Challenges..

[41] Al-Jaroodi J, Mohamed N, Abukhousa E (2020). Health 4.0. IEEE Access.

[42] Li J, Carayon P. Health Care 4.0: A Vision for Smart and Connected Health Care. IISE Trans Healthc Syst Eng. 2021;1–10. 10.1080/24725579.2021.1884627. 10.1080/24725579.2021.1884627 PMC8423174 34497970

[43] Embracing Healthcare 4.0. https://www.siemens-healthineers.com/insights/news/embracing-healthcare-4-0.html. Accessed 30 Mar 2022.

[44] Hathaliya JJ, Tanwar S, Tyagi S, Kumar N (2019). Securing Electronics Healthcare Records in Healthcare 4.0 : A Biometric-Based Approach. Comput Electr Eng..

[45] Schmidt JV, McCartney PR (2000). History and Development of Fetal Heart Assessment. J Obstet Gynecol Neonatal Nurs..

[46] Alfirevic Z, Gyte GM, Cuthbert A, Devane D. Continuous cardiotocography (CTG) as a form of electronic fetal monitoring (EFM) for fetal assessment during labour. Cochrane Database Syst Rev. 2019;2019(5). 10.1002/14651858.CD006066.pub3. 10.1002/14651858.CD006066 16856111

[47] Smith VJ, Marshall A, Lie M, Bidmead E, Beckwith B, Van Oudgaarden E (2021). Implementation of a fetal ultrasound telemedicine service: women’s views and family costs. BMC Pregnancy Childbirth..

[48] Mhajna M, Schwartz N, Levit-Rosen L, Warsof S, Lipschuetz M, Jakobs M (2020). Wireless, Remote Solution for Home Fetal and Maternal Heart Rate Monitoring. Am J Obstet Gynecol MFM..

[49] Al-Turjman F, Nawaz MH, Ulusar UD (2020). Intelligence in the Internet of Medical Things era. Comput Commun..

[50] Cömert Z, Kocamaz A. A Study of Artificial Neural Network Training Algorithms for Classification of Cardiotocography Signals. Bitlis Eren Univ J Sci Technol. 2017;7(2):93–103. 10.17678/beuscitech.338085.

[51] Marzbanrad F, Stroux L, Clifford GD (2018). Cardiotocography and beyond: A Review of One-Dimensional Doppler Ultrasound Application in Fetal Monitoring. Physiol Meas..

[52] Kovács F, Horváth C, Balogh ÁT, Hosszú G (2011). Fetal Phonocardiography—Past and Future Possibilities. Comput Methods Programs Biomed..

[53] Várady P, Wildt L, Benyó Z, Hein A (2003). An Advanced Method in Fetal Phonocardiography. Comput Methods Prog Biomed..

[54] Maulik D, Nanda NC, Maulik D, Vilchez G (2017). A Brief History of Fetal Echocardiography and Its Impact on the Management of Congenital Heart Disease. Echocardiography (Mount Kisco, NY)..

[55] Lee MY, Won HS (2013). Technique of Fetal Echocardiography. Obstet Gynecol Sci..

[56] Satomi G (2015). Guidelines for Fetal Echocardiography: Guidelines for Fetal Echocardiography. Pediatr Int..

[57] Peters MJ, Stinstra JG, Uzunbajakau S, Srinivasan N. Fetal Magnetocardiography. In: Lin JC, editor. Advances in Electromagnetic Fields in Living Systems. vol. 4. New York: Springer-Verlag; 2005. p. 1–40. 10.1007/0-387-24024-1_1.

[58] Strasburger JF, Cheulkar B, Wakai RT (2008). Magnetocardiography for Fetal Arrhythmias. Heart Rhythm Off J Heart Rhythm Soc..

[59] Quartero HWP, Stinstra JG, Golbach EGM, Meijboom EJ, Peters MJ (2002). Clinical Implications of Fetal Magnetocardiography: Clinical Use of Fetal MCG. Ultrasound Obstet Gynecol..

[60] Abdulhay EW, Oweis RJ, Alhaddad AM, Sublaban FN, Radwan MA, Almasaeed HM (2014). Non-Invasive Fetal Heart Rate Monitoring Techniques: Review Article. Biomed Sci Eng..

[61] Kahankova R, Martinek R, Jaros R, Behbehani K, Matonia A, Jezewski M (2020). A Review of Signal Processing Techniques for Non-Invasive Fetal Electrocardiography. IEEE Rev Biomed Eng..

[62] Hamelmann P, Kolen A, Schmitt L, Vullings R, van Assen H, Mischi M, et al. Ultrasound Transducer Positioning Aid for Fetal Heart Rate Monitoring. In: 2016 38th Annual International Conference of the IEEE Engineering in Medicine and Biology Society (EMBC). Orlando: IEEE; 2016. p. 4105–4108. 10.1109/EMBC.2016.7591629. 10.1109/EMBC.2016.7591629 28269185

[63] Cohen WR, Hayes-Gill B (2014). Influence of maternal body mass index on accuracy and reliability of external fetal monitoring techniques. Acta Obstet Gynecol Scand..

[64] Sameni. A Review of Fetal ECG Signal Processing Issues and Promising Directions. Open Pacing Electrophysiol Ther J. 2010. 10.2174/1876536X01003010004. 10.2174/1876536X01003010004 PMC3100207 21614148

[65] Okada DM, Chow AW, Bruce VT (1977). Neonatal Scalp Abscess and Fetal Monitoring: Factors Associated with Infection. Am J Obstet Gynecol..

[66] Graatsma E, Miller J, Mulder E, Harman C, Baschat A, Visser G (2010). Maternal Body Mass Index Does Not Affect Performance of Fetal Electrocardiography. Am J Perinatol..

[67] Barnova K, Martinek R, Jaros R, Kahankova R, Matonia A, Jezewski M (2021). A Novel Algorithm Based on Ensemble Empirical Mode Decomposition for Non-Invasive Fetal ECG Extraction. PLoS ONE..

[68] Jaros R, Martinek R, Kahankova R, Koziorek J (2019). Novel Hybrid Extraction Systems for Fetal Heart Rate Variability Monitoring Based on Non-Invasive Fetal Electrocardiogram. IEEE Access Pract Innovations Open Solutions..

[69] Barnova K, Martinek R, Jaros R, Kahankova R, Behbehani K, Snasel V (2021). System for Adaptive Extraction of Non-Invasive Fetal Electrocardiogram. Appl Soft Comput..

[70] Gobillot S, Fontecave-Jallon J, Equy V, Rivet B, Gumery PY, Hoffmann P (2018). Non-Invasive Fetal Monitoring Using Electrocardiography and Phonocardiography: A Preliminary Study. J Gynecol Obstet Hum Reprod..

[71] Castillo E, Morales DP, García A, Parrilla L, Ruiz VU, Álvarez-Bermejo JA (2018). A Clustering-Based Method for Single-Channel Fetal Heart Rate Monitoring. PLoS ONE..

[72] Gurve D, Krishnan S (2020). Separation of Fetal-ECG From Single-Channel Abdominal ECG Using Activation Scaled Non-Negative Matrix Factorization. IEEE J Biomed Health Inform..

[73] Da Poian G, Bernardini R, Rinaldo R (2016). Separation and Analysis of Fetal-ECG Signals From Compressed Sensed Abdominal ECG Recordings. IEEE Trans Biomed Eng..

[74] Signorini M, Lanzola G, Torti E, Fanelli A, Magenes G (2018). Antepartum Fetal Monitoring through a Wearable System and a Mobile Application. Technologies..

[75] Corona-Figueroa A (2019). A Portable Prototype for Diagnosing Fetal Arrhythmia. Inform Med Unlocked..

[76] Yuan L, Yuan Y, Zhou Z, Bai Y, Wu S (2019). A Fetal ECG Monitoring System Based on the Android Smartphone. Sensors..

[77] Rashkovska A, Avbelj V. Abdominal Fetal ECG Measured with Differential ECG Sensor. In: 2017 40th International Convention on Information and Communication Technology, Electronics and Microelectronics (MIPRO). Opatija: IEEE; 2017. p. 289–291. 10.23919/MIPRO.2017.7973436.

[78] Theodoridou A. Current Methods of Non-Invasive Fetal Heart Rate Surveillance. Clinical and Experimental Obstetrics & Gynecology. 2020;47(4):459. 10.31083/j.ceog.2020.04.5422.

[79] Kapaya H, Dimelow ER, Anumba D (2018). Women’s Experience of Wearing a Portable Fetal-Electrocardiogram Device to Monitor Small-for-Gestational Age Fetus in Their Home Environment. Womens Health..

[80] GE Healthcare Systems. https://www.gehealthcare.com. Accessed 30 Mar 2022.

[81] Mindchild. https://www.mindchild.com. Accessed 30 Mar 2022.

[82] Sulas E, Urru M, Tumbarello R, Raffo L, Sameni R, Pani D (2021). A Non-Invasive Multimodal Foetal ECG–Doppler Dataset for Antenatal Cardiology Research. Sci Data..

[83] Nemo Healthcare. https://nemohealthcare.com. Accessed 30 Mar 2022.

[84] Vullings R, van Laar JOEH (2020). Non-Invasive Fetal Electrocardiography for Intrapartum Cardiotocography. Front Pediatr..

[85] Avalon beltless feotal monitoring solution. Philips. Available: https://www.philips.co.uk/healthcare/product/HC866488/avalon-beltless-feotal-monitoring-solution. Accessed 1 Jan 2022.

[86] Martinek R, Kahankova R, Nazeran H, Konecny J, Jezewski J, Janku P (2017). Non-Invasive Fetal Monitoring: A Maternal Surface ECG Electrode Placement-Based Novel Approach for Optimization of Adaptive Filter Control Parameters Using the LMS and RLS Algorithms. Sensors..

[87] Aggarwal G, Wei Y (2021). Non-Invasive Fetal Electrocardiogram Monitoring Techniques: Potential and Future Research Opportunities in Smart Textiles. Signals..

[88] Kubicek J, Fiedorova K, Vilimek D, Cerny M, Penhaker M, Janura M, et al. Recent Trends, Construction and Applications of Smart Textiles and Clothing for Monitoring of Health Activity: A Comprehensive Multidisciplinary Review. IEEE Rev Biomed Eng. 2020;1. 10.1109/RBME.2020.3043623. 10.1109/RBME.2020.3043623 33301410

[89] Stoppa M, Chiolerio A (2014). Wearable Electronics and Smart Textiles: A Critical Review. Sensors..

[90] Zarzoso V, Nandi AK (2001). Noninvasive Fetal Electrocardiogram Extraction: Blind Separation versus Adaptive Noise Cancellation. IEEE Trans Biomed Eng..

[91] Martín-Clemente R, Camargo-Olivares JL, Hornillo-Mellado S, Elena M, Román I (2011). Fast Technique for Noninvasive Fetal ECG Extraction. IEEE Trans Biomed Eng..

[92] Ghobadi Azbari P, Mohaqeqi S, Ghanbarzadeh Gashti N, Mikaili M (2016). Introducing a Combined Approach of Empirical Mode Decomposition and PCA Methods for Maternal and Fetal ECG Signal Processing. J Matern-Fetal Neonatal Med..

[93] Wu S, Shen Y, Zhou Z, Lin L, Zeng Y, Gao X (2013). Research of Fetal ECG Extraction Using Wavelet Analysis and Adaptive Filtering. Comput Biol Med..

[94] Assaleh K (2007). Extraction of Fetal Electrocardiogram Using Adaptive Neuro-Fuzzy Inference Systems. IEEE Trans Biomed Eng..

[95] Liu Sj, Liu Dl, Zhang Jq, Zeng Yj. Extraction of Fetal Electrocardiogram Using Recursive Least Squares and Normalized Least Mean Squares Algorithms. In: 2011 3rd International Conference on Advanced Computer Control. Harbin: IEEE; 2011. p. 333–336. 10.1109/ICACC.2011.6016426.

[96] Alexander ST. Fast Transversal Filters. In: Adaptive Signal Processing. New York: Springer New York; 1986. p. 154–176. 10.1007/978-1-4612-4978-8_11.

[97] Martinek R, Kahankova R, Jaros R, Barnova K, Matonia A, Jezewski M (2021). Non-Invasive Fetal Electrocardiogram Extraction Based on Novel Hybrid Method for Intrapartum ST Segment Analysis. IEEE Access Pract Innovations Open Solutions..

[98] Martinek R, Barnova K, Jaros R, Kahankova R, Kupka T, Jezewski M (2020). Passive Fetal Monitoring by Advanced Signal Processing Methods in Fetal Phonocardiography. IEEE Access Pract Innovations Open Solutions..

[99] Mhajna M, Sadeh B, Yagel S, Sohn C, Schwartz N, Warsof S (2022). A novel, cardiac-derived algorithm for uterine activity monitoring in a wearable remote device. Front Bioeng Biotechnol.

[100] de Campos DA, Arulkumaran S (2015). FIGO consensus guidelines on intrapartum fetal monitoring. Int J Gynecol Obstet..

[101] Bergstra J, Bengio Y (2012). Random search for hyper-parameter optimization. J Mach Learn Res.

[102] Kennedy J, Eberhart R. Particle Swarm Optimization. In: Proceedings of ICNN’95 - International Conference on Neural Networks. vol. 4. Perth: IEEE; 1995. p. 1942–1948. 10.1109/ICNN.1995.488968.

[103] Storn R, Price K (1997). Differential Evolution - A Simple and Efficient Heuristic for global Optimization over Continuous Spaces. J Glob Optim..

[104] Mirjalili S (2015). Moth-Flame Optimization Algorithm: A Novel Nature-Inspired Heuristic Paradigm. Knowl-Based Syst..

[105] Nasiri M. Fetal Electrocardiogram Signal Extraction by ANFIS Trained with PSO Method. Int J Electr Comput Eng (IJECE). 2012;2(2):247–260. 10.11591/ijece.v2i2.231.

[106] Elmansouri K, Latif R, Maoulainine F (2014). Improvement of Fetal Electrocardiogram Extraction by Application of Fuzzy Adaptive Resonance Theory to Adaptive Neural Fuzzy System. Int J Innov Appl Stud..

[107] Panigrahy D, Rakshit M, Sahu PK. An Efficient Method for Fetal ECG Extraction from Single Channel Abdominal ECG. In: 2015 International Conference on Industrial Instrumentation and Control (ICIC). Pune: IEEE; 2015. p. 1083–1088. 10.1109/IIC.2015.7150908.

[108] Kockanat S, Kockanat S. Analysis and Extraction of Fetal Electrocardiogram Signal with Adaptive Filtering Using Differential Evolution Algorithm. Cumhuriyet Sci J. 2018;294–302. 10.17776/csj.407424.

[109] Panigrahy D, Sahu PK (2017). Extraction of Fetal ECG Signal by an Improved Method Using Extended Kalman Smoother Framework from Single Channel Abdominal ECG Signal. Australas Phys Eng Sci Med..

[110] Jibia MS, Jibia AU. Fetal Electrocardiogram Extraction Using Moth Flame Optimization (MFO)-Based Adaptive Filter. Adv Sci Technol Eng Syst J. 2021;6(2):303–312. 10.25046/aj060235.

[111] Silwattananusarn T, Kanarkard W, Tuamsuk K (2016). Enhanced Classification Accuracy for Cardiotocogram Data with Ensemble Feature Selection and Classifier Ensemble. J Comput Commun..

[112] Subasi A, Kadasa B, Kremic E (2020). Classification of the Cardiotocogram Data for Anticipation of Fetal Risks Using Bagging Ensemble Classifier. Procedia Comput Sci..

[113] Krupa N, Ma M, Zahedi E, Ahmed S, Hassan FM (2011). Antepartum Fetal Heart Rate Feature Extraction and Classification Using Empirical Mode Decomposition and Support Vector Machine. Biomed Eng Online..

[114] Carter EB, Tuuli MG, Caughey AB, Odibo AO, Macones GA, Cahill AG (2016). Number of Prenatal Visits and Pregnancy Outcomes in Low-Risk Women. J Perinatol..

[115] Laranjo L, Dunn AG, Tong HL, Kocaballi AB, Chen J, Bashir R (2018). Conversational Agents in Healthcare: A Systematic Review. J Am Med Inform Assoc..

[116] Mutabazi E, Ni J, Tang G, Cao W (2021). A Review on Medical Textual Question Answering Systems Based on Deep Learning Approaches. Appl Sci..

[117] Abdallah A, Kasem M, Hamada MA, Sdeek S. Automated Question-Answer Medical Model Based on Deep Learning Technology. In: Proceedings of the 6th International Conference on Engineering & MIS 2020. Almaty Kazakhstan: ACM; 2020. p. 1–8. 10.1145/3410352.3410744.

[118] Nadarzynski T, Miles O, Cowie A, Ridge D (2019). Acceptability of Artificial Intelligence (AI)-Led Chatbot Services in Healthcare: A Mixed-Methods Study. Digital Health..

[119] Adamopoulou E, Moussiades L (2020). Chatbots: History, Technology, and Applications. Mach Learn Appl..

[120] Philip P, Micoulaud-Franchi JA, Sagaspe P, Sevin ED, Olive J, Bioulac S (2017). Virtual Human as a New Diagnostic Tool, a Proof of Concept Study in the Field of Major Depressive Disorders. Sci Rep..

[121] Lucas GM, Rizzo A, Gratch J, Scherer S, Stratou G, Boberg J (2017). Reporting Mental Health Symptoms: Breaking Down Barriers to Care with Virtual Human Interviewers. Front Robot AI..

[122] Levin E, Levin A (2006). Evaluation of Spoken Dialogue Technology for Real-Time Health Data Collection. J Med Internet Res..

[123] Giorgino T, Azzini I, Rognoni C, Quaglini S, Stefanelli M, Gretter R (2005). Automated Spoken Dialogue System for Hypertensive Patient Home Management. Int J Med Inform..

[124] Azzini I, Falavigna D, Giorgino T, Gretter R, Quaglini S, Rognoni C, et al. Automated spoken dialog system for home care and data acquisition from chronic patients. In: The New Navigators: From Professionals to Patients. Amsterdam: IOS Press; 2003. p. 146–51. 14663978

[125] Black LA, McTear M, Black N, Harper R, Lemon M. Appraisal of a Conversational Artefact and Its Utility in Remote Patient Monitoring. In: 18th IEEE Symposium on Computer-Based Medical Systems (CBMS’05). Dublin: IEEE; 2005. p. 506–508. 10.1109/CBMS.2005.33.

[126] Harper R, Nicholl P, McTear M, Wallace J, Black LA, Kearney P. Automated Phone Capture of Diabetes Patients Readings with Consultant Monitoring via the Web. In: 15th Annual IEEE International Conference and Workshop on the Engineering of Computer Based Systems (Ecbs 2008). Belfast: IEEE; 2008. p. 219–226. 10.1109/ECBS.2008.31.

[127] Griol D, Carbó J, Molina JM (2013). An Automatic Dialog Simulation Technique to Develop and Evaluate Interactive Conversational Agents. Appl Artif Intell..

[128] Battineni G, Chintalapudi N, Amenta F (2020). AI Chatbot Design during an Epidemic like the Novel Coronavirus. Healthcare..

[129] Rodsawang C, Thongkliang P, Intawong T, Sonong A, Thitiwatthana Y, Chottanapund S (2020). Designing a competent chatbot to counter the Covid-19 pandemic and empower risk communication in an emergency response system. OSIR J.

[130] Dennis AR, Kim A, Rahimi M, Ayabakan S (2020). User Reactions to COVID-19 Screening Chatbots from Reputable Providers. J Am Med Inform Assoc..

[131] Walwema J, The WHO (2021). Health Alert: Communicating a Global Pandemic with WhatsApp. J Bus Tech Commun..

[132] Almalki M, Azeez F (2020). Health Chatbots for Fighting COVID-19: A Scoping Review. Acta Inform Med..

[133] Chung K, Cho HY, Park JY (2021). A Chatbot for Perinatal Women’s and Partners’ Obstetric and Mental Health Care: Development and Usability Evaluation Study. JMIR Med Inform..

[134] Vaira L, Bochicchio MA, Conte M, Casaluci FM, Melpignano A. MamaBot: A System Based on ML and NLP for Supporting Women and Families during Pregnancy. In: Proceedings of the 22nd International Database Engineering & Applications Symposium on - IDEAS 2018. Villa San Giovanni: ACM Press; 2018. p. 273–277. 10.1145/3216122.3216173.

[135] Marko KI, Ganju N, Krapf JM, Gaba ND, Brown JA, Benham JJ (2019). A Mobile Prenatal Care App to Reduce In-Person Visits: Prospective Controlled Trial. JMIR mHealth uHealth..

[136] Lavariega JC, Córdova GA, Gómez LG, Avila A. Monitoring and Assisting Maternity-Infant Care in Rural Areas (Mamicare). In: E-Health and Telemedicine: Concepts, Methodologies, Tools, and Applications. Pennsylvania: IGI Global; 2016. p. 347–59.

[137] Razaque A, Amsaad F, Jaro Khan M, Hariri S, Chen S, Siting C (2019). Survey: Cybersecurity Vulnerabilities, Attacks and Solutions in the Medical Domain. IEEE Access Pract Innovations Open Solutions..

